# Porcupine Inhibitor LGK974 Downregulates the Wnt Signaling Pathway and Inhibits Clear Cell Renal Cell Carcinoma

**DOI:** 10.1155/2020/2527643

**Published:** 2020-02-13

**Authors:** Jianyi Li, Guangzhen Wu, Yingkun Xu, Jiatong Li, Ningke Ruan, Yougen Chen, Qi Zhang, Qinghua Xia

**Affiliations:** ^1^Department of Urology, Shandong Provincial Hospital Affiliated to Shandong University, Jinan, China; ^2^Department of Urology, The First Affiliated Hospital of Dalian Medical University, Dalian, China; ^3^Department of Geriatrics, Shandong Provincial Hospital Affiliated to Shandong First Medical University & Shandong Academy of Medical Sciences, Jinan, China; ^4^The Nursing College of Zhengzhou University, Zhengzhou, China

## Abstract

Targeted therapy for kidney cancer has achieved significant clinical results. However, because most patients who use targeted therapy will develop drug resistance, we still need to constantly explore new therapeutic targets. Although porcupine (PORCN) as a palmitoyltransferase plays a crucial role in the activation and secretion of Wnt proteins and affects the activity of the Wnt signaling pathway, little is known about the role of PORCN in clear cell renal cell carcinoma (ccRCC). We found that PORCN is highly expressed in renal cancer cell lines and patients with renal cell carcinoma with high expression of PORCN have a poor prognosis. Pathway analysis of PORCN and its related proteins showed that PORCN played a role through the Wnt signaling pathway, and there was a strong coexpression relationship between PORCN and Wnt proteins. Therefore, PORCN may be a potential and effective target for ccRCC. In the present study, we found that LGK974 could inhibit proliferation and colony formation and induce apoptosis in ccRCC cells. We also found that LGK974 could inhibit the migration and invasion of renal cell carcinoma and reduce the expression of mesenchymal markers. After treatment with LGK974, the expression level of *β*-catenin, a key protein in the classical Wnt pathway, was significantly decreased, and the expression levels of the target genes cyclin D1, c-Myc, MMP9, and MMP2 in the Wnt signaling pathway were also significantly decreased, which represented a significant decrease in the activity of the Wnt signaling pathway. At the same time, the cycle of renal cancer cells was significantly blocked. In conclusion, our results indicate that LGK974 could significantly inhibit the progression of renal cancer cells in a safe concentration range, so PORCN may be a safe and effective target for patients with renal cancer.

## 1. Introduction

Renal cell carcinoma (RCC) accounts for 2-3% of all cancers, and the highest incidence is in Western countries [1]. According to the Cancer Statistics of the USA in 2019, it is estimated that 73,820 new cases of kidney cancer will be diagnosed and 14,770 patients will die of kidney cancer [2]. Compared with the data of 2018 (65340 new cases and 14970 deaths) [3], the number of new cases is increasing and the number of deaths has not decreased significantly though the diagnosis and treatment techniques of kidney cancer have been improving. According to the China Cancer Statistics 2015, it is expected that there will be 66,800 new cases of kidney cancer, and 23,400 patients will die of kidney cancer in 2015 [4]. Therefore, whether in Western countries or China, the impact of kidney cancer on human health cannot be underestimated.

Clear cell renal cell carcinoma (ccRCC) accounts for 75–80% of renal cell carcinoma (RCC) cases and is the most common subtype of RCC [5]. It is reported that 20–30% of RCC patients have local or distant metastases at diagnosis, and most patients with advanced RCC do not respond well to chemotherapy or radiotherapy [6]. In the clinic, many kidney cancer patients respond well to targeted drugs. Although the targeted drugs for the treatment of RCC have a certain therapeutic effect on advanced RCC, due to the problem of drug resistance, they are still unsatisfactory. Therefore, exploring and applying new therapeutic targets will benefit more patients.

Studies have shown that the Wnt signaling pathway is involved in many biological processes of cancer cell development, including the initiation, growth, differentiation, metastasis, senescence, and death of cancer cells [7]. According to reports, the Wnt signaling pathway is also involved in the development of RCC [8]. To our knowledge, according to whether *β*-catenin is involved in signal transduction, the Wnt signaling pathway can be divided into canonical or noncanonical pathways [8]. Based on the previous research, both canonical [9–11] and noncanonical Wnt signaling pathways [12, 13] could affect the progression of kidney cancer. Previous studies have focused solely on classical or noncanonical Wnt signaling pathways, and few have been able to suppress both pathways. To explore the effects of inhibiting the two pathways on renal cancer, we have noted the PORCN gene. Palmitoylation of Wnt ligand by PORCN is a very important step in its secretion and activation [14], this process makes PORCN an ideal therapeutic target leading to the development of LGK974, an effective, selective small molecule PORCN inhibitor [15]. We aimed at investigating the effects of PORCN inhibitor LGK974 on the biological behaviors of proliferation, apoptosis, migration, and invasion of renal cancer cells.

## 2. Materials and Methods

### 2.1. Gene Ontology Analysis and KEGG Pathway Analysis

WebGestalt [16] (http://www.webgestalt.org/) online tool (Annotation, Visualization, and Integrated Discovery database) is used for GO (Gene Ontology) enrichment analysis and KEGG pathway analysis. KEGG, Panther, and Reactome pathway analysis of PORCN and its 30 most related genes were completed by KOBAS online tool [17] (http://kobas.cbi.pku.edu.cn/kobas3) and visualized by R language.

### 2.2. Construction of Protein-Protein Interaction Network, Gene Mutation Analysis, and Coexpression Analysis

The String online tool [18] (https://string-db.org/cgi/) was used to study the relationship between various genes. Analysis of gene mutations and coexpression was performed on the cBioportal online tool [19, 20] (https://www.cbioportal.org/).

### 2.3. Cell Counting Kit 8 Assay

Cell Counting Kit-8 was used to evaluate the proliferation ability of RCC cell lines (Dojindo, Japan). The appropriate number of cells were inoculated in a 96-well plate, and after a certain period of treatment, 10 *μ*l of CCK-8 was added to the corresponding wells and incubated with cells for 1 hour in a 5% CO_2_ incubator. The optical density (OD) of each well was measured with a microplate reader at a wavelength of 450 nm.

### 2.4. Clone Formation Assay

Appropriate cells were inoculated in 6-well plates and treated with the LGK974 for 10 days. When a clone was visible to the naked eye in a culture dish, the culture was terminated. Next, the cells were washed twice with PBS, fixed with 4% formaldehyde, and stained with crystal violet (MCE, B0324A). The number of colonies was counted after rinsing with double distilled water.

### 2.5. Flow Cytometry

For detecting apoptosis, ACHN and 786-O cells were collected and washed twice with precooled phosphate-buffered saline (PBS). 500 mg binding buffer was used to resuspend these cells, followed by the addition of 5 *μ*l of Annexin V-FITC and 5 *μ*l of propyl propionate (BD, 559763). These cells were incubated under the condition of avoiding light at room temperature for 15 minutes and then analyzed by flow cytometry (BD, NJ, USA). At least 10,000 cells were collected. The results were analyzed by FlowJo 7.6.2 software (BD, USA). For observing the cell cycle, ACHN and 786-O cells were collected and washed with PBS buffer. The cells were then resuspended in precooled 75% ethanol and spent the night at 4°C. Then the cells were washed twice with PBS and resuspended in deionized water containing 1 mg/mL RNase A and incubated at 37°C for 40 min. The cells were dyed with PI staining solution for 20 minutes in the dark. All the samples were analyzed through flow cytometry (BD Biosciences, Franklin Lakes, NJ, USA).

### 2.6. Wound Healing Assay

ACHN and 786-O cells were plated in 6-well plates and cultured with a certain concentration of LGK974. When confluent cell layers reached approximately 95%, the 1000 *μ*L sterile pipette tip was used to scratch it. Then, PBS was applied to wash each well. The cells were then cultured in a serum-free medium containing a specified concentration of LGK974. The incubation time of each cell line varies according to the dynamics of individual cell migration. The healing rate of cells was observed after a certain period of time. The healing rate was assessed by Image-Pro Plus.

### 2.7. Transwell Assay

After LGK974 was applied to ACHN and 786-O cells with a certain concentration for 72 h, the cells were added into the upper cup of transwell chambers (24-well, 8 *μ*M pore membrane; Corning Incorporated, NY, USA). For cell invasion, transwell chamber was coated with Matrigel (Corning Incorporated, NY), not for cell migration. 500 *μ*L of medium containing 10% FBS was added to the lower chamber. After incubation at 37°C for 24 h (786-O) or 36°h (ACHN), the cells above the chamber were wiped with a cotton swab. Cells on the lower side of the membrane were fixed in 4% paraformaldehyde and stained with crystal violet. The images were then taken in a randomly selected area by a microscope (Leica Microsystems, GmbH).

### 2.8. RNA Extraction and Real-Time PCR

TRIzol reagent was used to extract total RNA (Takara, 9108). The Quantscript RT Kit was used to synthesize cDNA from 2 *μ*g RNA (Takara, PR047A) according to the manufacturer's instructions. The primer sequences used for real-time PCR were as follows:

  PORCN-F, 5′-CTTTGGAGCTCTGGCCATCTTC-3′
  PORCN-R, 5′-CCAGCTGAGCTCTGACCACTTG-3′
  MMP2-F, 5′-CTCATCGCAGATGCCTGGAA-3′
  MMP2-R, 5′-TTCAGGTAATAGGCACCCTTGAAGA-3′
  MMP9-F, 5′-ACGCACGACGTCTTCCAGTA-3′
  MMP9-R, 5′-CCACCTGGTTCAACTCACTCC-3′

### 2.9. Western Blotting

The protein of ACHN and 786-O was extracted by RIPA lysis buffer (CST, 9806), and protein concentration was detected by the BCA method (Abcam, ab102536). Each sample (30 *μ*g protein) was separated by 8–12% SDS-PAGE gel (Keygen Biotech, KGP113K), then transferred to PVDF membrane (Millipore, R8CA8257B), blocked with 5% skim milk powder for 1 hour, and incubated overnight with primary antibody. The membrane was then incubated with peroxidase-conjugated secondary antibody, and the immunoreactive bands were observed by the ECL system.

### 2.10. Immunohistochemistry

ccRCC and normal specimens were fixed with 4% paraformaldehyde and embedded in paraffin for slicing. Antigen retrieval was performed with 10 mM citrate buffer (pH6.0, 13 min). In order to label, the sections were incubated with a rabbit polyclonal antibody (anti-PORCN) at 4°C overnight. The sections were incubated with the second antibody for 30 minutes and detected by DAB kit (Abcam, ab64238). According to the manufacturer's procedures, the nucleus was stained with hematoxylin (Abcam, ab143166). The results were evaluated by two independent pathologists.

### 2.11. Statistics

The data are expressed as the mean ± SEM. Statistical analysis was performed using Prism software (GraphPad, CA, USA). Statistical significance of differences between and among groups was assessed using *t*-test and one-way ANOVA, respectively. Significant differences are indicated as follows: ^*∗*^*p* < 0.05; ^*∗∗*^*p* < 0.01; ^*∗∗∗*^*p* < 0.001.

## 3. Results

### 3.1. PORCN Is Highly Expressed in Renal Cancer Cell Lines and Is Associated with Poor Prognosis

**Figure 1:**
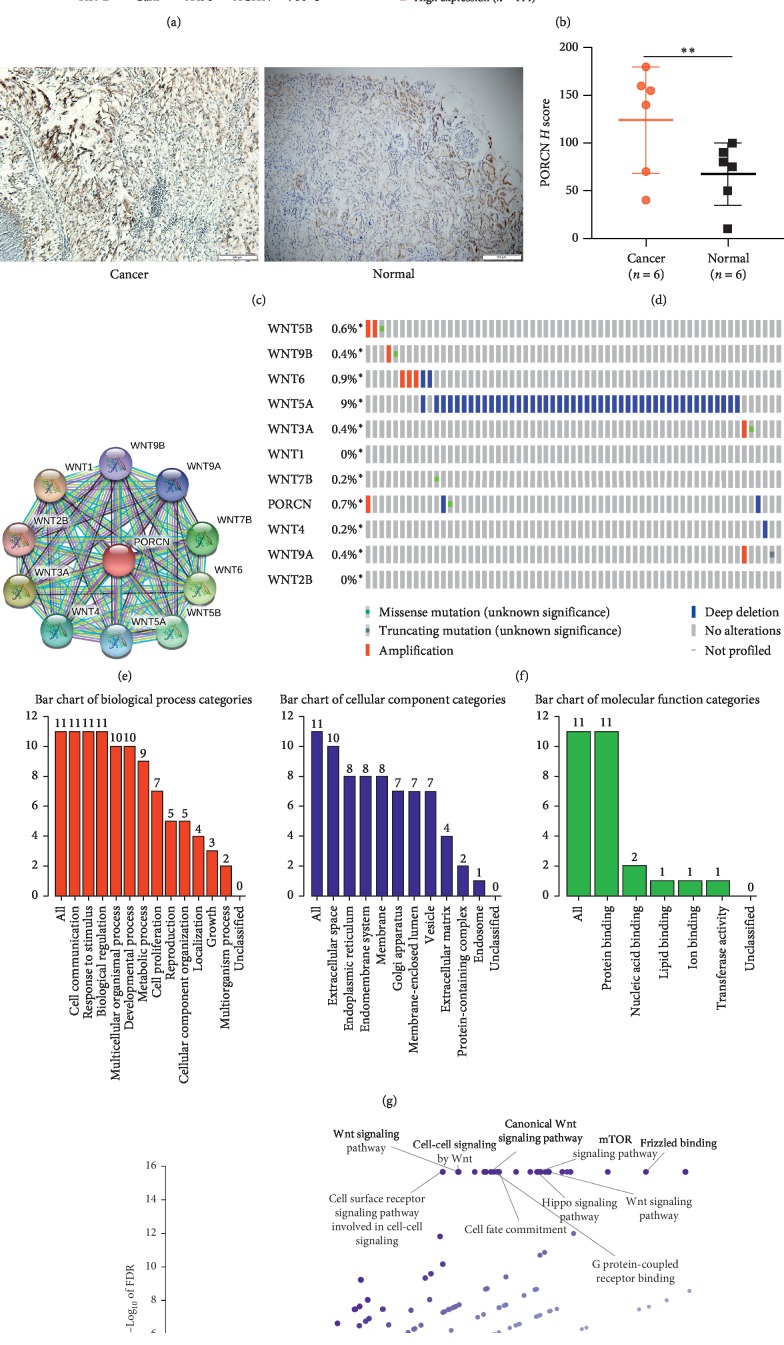
Bioinformatics analysis related to PORCN. (a) Real-time PCR was used to detect the expression of PORCN in five cell lines. (b) Survival analysis of patients with renal cell carcinoma with high and low expression of PORCN by the Human Protein Atlas online tool. (c, d) The expression of PORCN in RCC specimens. (e, f) PORCN and related proteins, as well as their mutations in kidney cancer. (g, h) GO analysis and KEGG analysis on PORCN and its related genes.

Although PORCN appears as an oncogene in other tumors, it is not known whether it is also an oncogene in renal cell carcinoma. We used real-time PCR to detect the expression of PORCN in four renal cancer cell lines 786-O, A498, ACHN, Caki, and normal renal epithelial cell lines HK-2. It was found that the expression of PORCN in 786-O, A498, and ACHN was significantly higher than that in HK-2; however, the expression level of PORCN in Caki was lower than that in HK-2 (Figure 1(a)). To investigate the role of PORCN in human ccRCC, we analyzed the expression of PORCN in 6 cancer tissues and adjacent tissues by immunohistochemistry. We found that the expression of PORCN in renal cell carcinoma was higher than that in normal tissue (Figures 1(c)–1(d)). Then we used the Human Protein Atlas online tool (https://www.proteinatlas.org/2019/9/26) to analyze the relationship between the expression of PORCN and prognosis (Figure 1(b)). From the survival analysis of 414 patients with high expression of PORCN and 114 patients with low expression of PORCN, we can see that the patients with high expression of PORCN have a poor prognosis.

### 3.2. The 10 Genes Most Relevant to PORCN, GO, and KEGG Pathway Analysis

To clarify which signaling pathway the PORCN works through, we first identified the top 10 genes that have a protein-protein interaction network with PORCN via the STRING website (Figure 1(c)). Gene mutations are often the cause of cancer. In order to determine whether PORCN and its related genes have mutations in renal cell carcinoma, we conducted a study using the cBioportal online tool. The results showed that in addition to Wnt1 and Wnt2b, PORCN and other related genes have varying degrees of mutation in renal cell carcinoma (Figure 1(d)), which may mean that PORCN and its related genes will play an important role in renal cell carcinoma. Next, we performed GO analysis and KEGG analysis on PORCN and its related genes. GO analysis shows that in the biological process, these genes are mainly concentrated in cell communication, response to stimulus, and biological regulation; in the cellular component, these genes are mainly enriched in extracellular space and endoplasmic reticulum; in molecular function among them, these genes are mainly concentrated in protein binding (Figure 1(e)). KEGG analysis shows that these genes were mainly enriched in the Wnt signaling pathway, cell-cell signaling by Wnt, canonical Wnt signaling pathway, TOR signaling pathway, etc (Figure 1(f)).

### 3.3. Protein-Protein Interaction and the Coexpression of PORCN and Wnt Proteins

**Figure 2:**
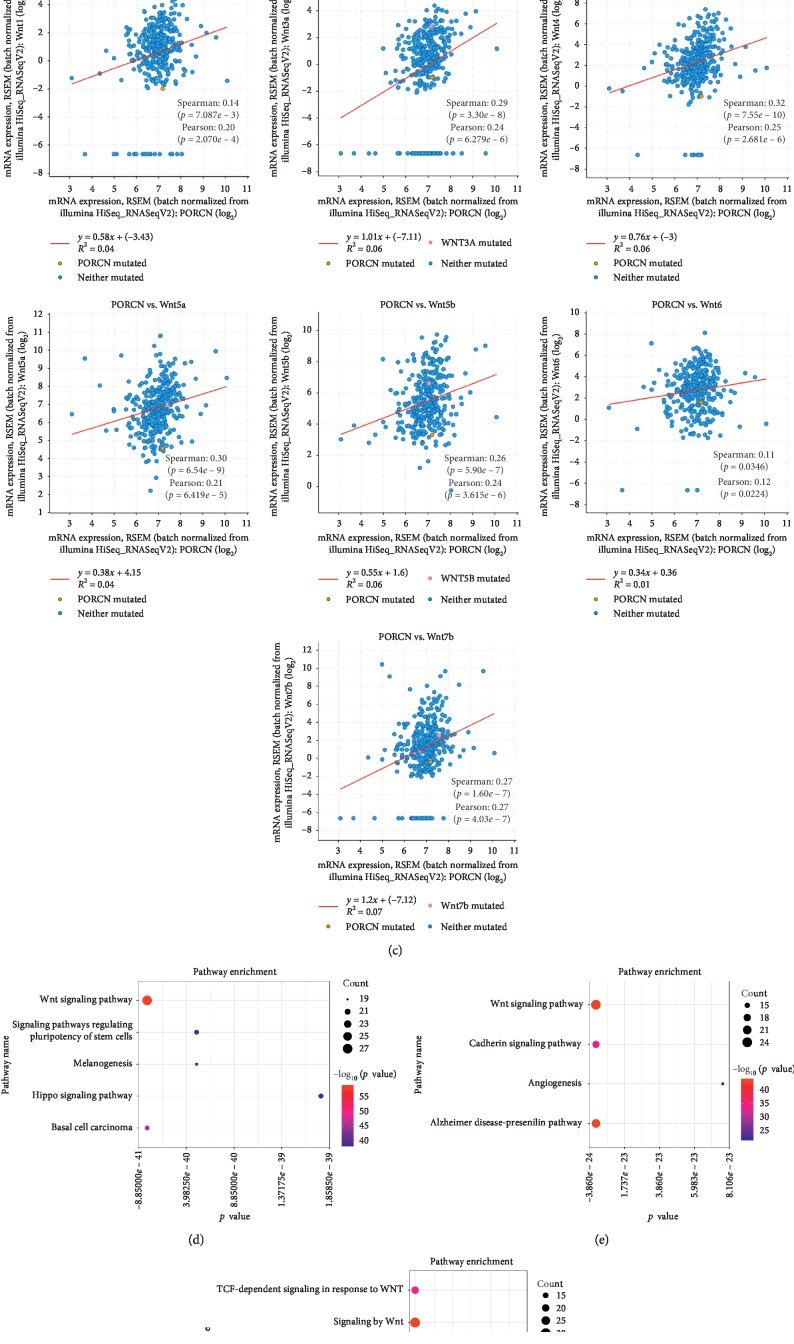
PORCN is coexpressed with Wnt protein and plays a role through Wnt signaling pathway. (a) Protein-protein interaction of PORCN and its related genes with Wnt signaling pathway-related proteins, apoptosis-related proteins, and EMT-related proteins. (b) PORCN and its related genes in the Wnt signaling pathway. (c) The coexpression of PORCN and Wnt proteins. (d–f) KEGG (d), Panther (e), and Reactome (f) pathway analysis of PORCN and its 30 most related genes.

In order to verify whether PORCN and its related genes play a role through the Wnt signaling pathway, we interacted PORCN and its related genes with Wnt signaling pathway-related proteins, apoptosis-related proteins, and EMT-related proteins (Figure 2(a)). It is found that there is a strong correlation between them, and then we verify the pathway through the DAVID online tool (DAVID, https://david.ncifcrf.gov/) [21] and find that they do exist in the Wnt signaling pathway (Figure 2(b)). Moreover, according to pathway analysis, PORCN and its related genes will affect the cell cycle. It is well known that coexpression between proteins often indicates that they can work together. We performed coexpression analysis of PORCN and its related genes through cBioportal online tools. The results showed that except Wnt2b, Wnt9a and Wnt9b, Wnt1, Wnt3a, Wnt4, Wnt5a, Wnt5b, Wnt6, and Wnt7b all had strong coexpression relationship with PORCN (Figure 2(c)). In order to further verify the relationship between PORCN and WNT signal pathways, we used the STRING website to find 30 genes most related to PORCN and then used KOBAS online tools for further pathway analysis, including KEGG (Figure 2(d)), Panther (Figure 2(e)), Reactome (Figure 2(f)), and visualization using R language. The results show that PORCN is closely related to WNT signal pathway.

### 3.4. LGK974 Kills ccRCC Cells in a Concentration- and Time-dependent Manner

**Figure 3:**
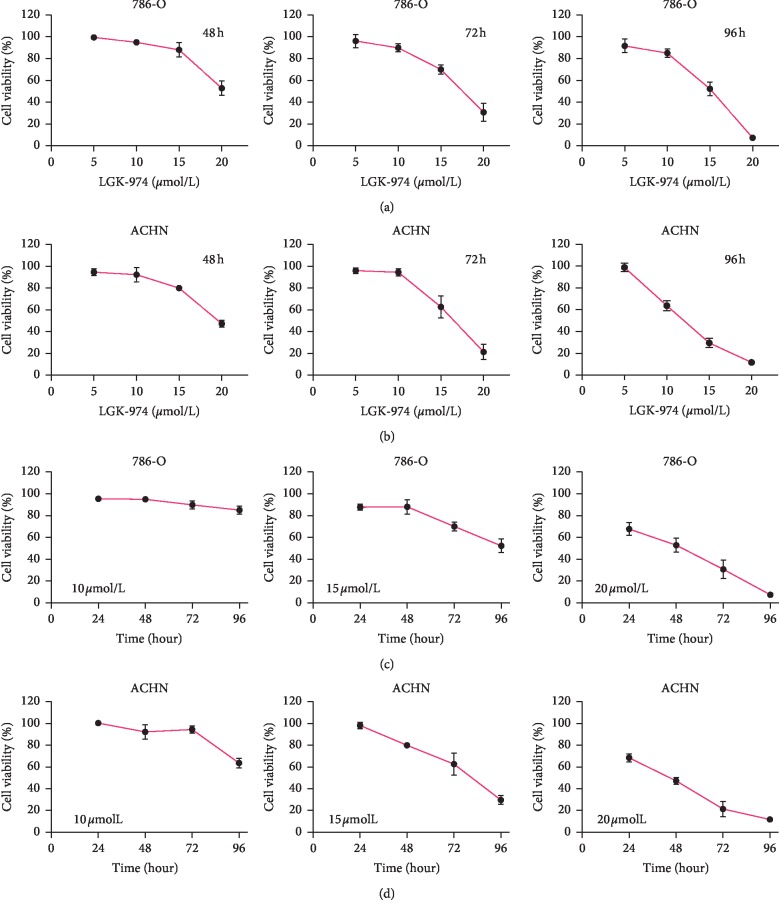
LGK974 kills ccRCC cells in a concentration- and time-dependent manner. (a) CCK8 assays were used to detect the difference in the viability of 786-O cells after 48, 72, and 96 h of treatment with different concentrations of LGK974. (b) CCK8 assays were used to detect the difference in the viability of ACHN cells after 48, 72, and 96 h of treatment with different concentrations of LGK974. (c) CCK8 assays were used to detect cell viability of 786-O cells at different times using the same concentration of LGK974 (10 *μ*M, 15 *μ*M, or 20 *μ*M). (d) CCK8 assays were used to detect cell viability of ACHN cells at different times using the same concentration of LGK974 (10 *μ*M, 15 *μ*M, or 20 *μ*M) ^*∗∗∗*^*p* < 0.001; ^*∗∗∗∗*^*p* < 0.0001.

To investigate how PORCN affects kidney cancer, we selected the PORCN inhibitor LGK974 for experiments. In order to study the killing effect of LGk974 on ccRCC cells, CCK8 experiments were carried out on ACHN and 786-O cell lines. When choosing anticancer drugs, safety should be the first. According to the research of Jun Liu et al. [15], LGK974 did not show major cytotoxicity when the concentration was as high as 20 *μ*M. We treated 786-O and ACHN cells with 5 *μ*M, 10 *μ*M, 15 *μ*M, 20 *μ*M LGK974, or vehicle control, respectively. We found that LGK974 significantly inhibits the growth of kidney cancer cells in a concentration-dependent manner (Figures 3(a) and 3(b)). To study whether LGK974 inhibited ccRCC cells in a time-dependent manner, we performed CCK8 experiments concerning previous drug concentrations at different drug action times (24, 48, 72, and 96 hours). We found that the killing effect of LGK974 increased with the increase of drug action time (Figures 3(c) and 3(d)). Our results showed that LGK974 kills ccRCC cells in a concentration-dependent and time-dependent manner.

### 3.5. LGK974 Inhibits Proliferation and Induces Apoptosis of ccRCC

**Figure 4:**
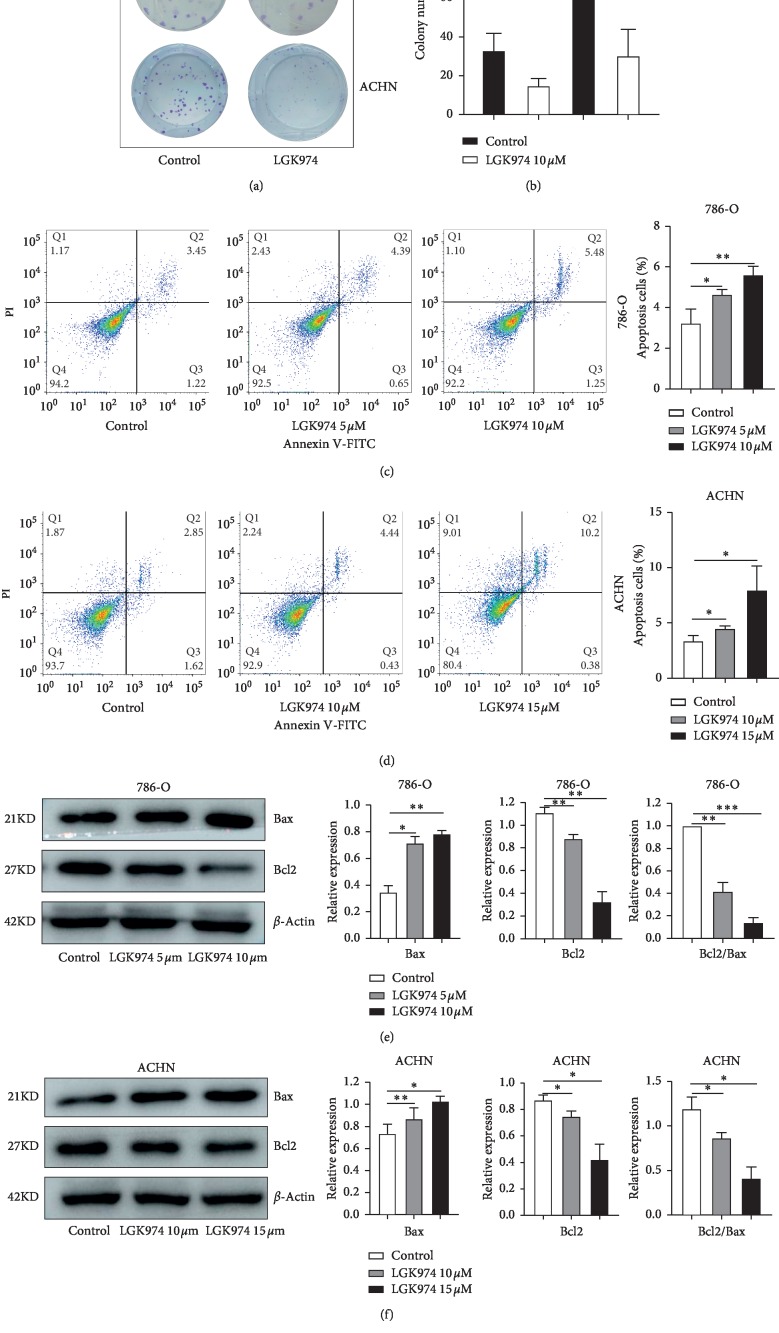
LGK974 inhibits renal cancer cell proliferation and induces apoptosis. (a, b) Colony formation assays: LGK974 (10 *μ*M) were applied to 786-O and ACHN cells for 96 h. (c, d) 786-O and ACHN cells were stained with Annexin V-FITC/PI and analyzed via flow cytometry. LGK974 (5 *μ*M or 10 *μ*M for 786-O cells and 10 *μ*M or 15* μ*M for ACHN cells) acted on the two cell lines. (e, f) Western blotting experiments were performed by treating renal cancer cells with DMSO, at different concentrations of LGK974 (786-O, 5 *μ*M or 10 *μ*M; ACHN, *10 μ*M or 15 *μ*M) for 72 h and then proteins were extracted. The protein levels of Bax and Bcl2 were determined via immunoblotting. All data are expressed as the mean ± SEM. The experiment was repeated at least three times. Statistical significance was determined using two-tailed Student's *t*-test or one-way ANOVA. ^*∗*^*p* < 0.05; ^*∗∗*^*p* < 0.01; ^*∗∗∗*^*p* < 0.001.

To study whether LGK974 inhibits the proliferative capacity of ccRCC, we performed colony formation assays with ACHN and 786-O cells. We observed a significant decrease in colony-forming ability of 786-O and ACHN cells after LGK974 treatment (Figures 4(a) and 4(b)). Then, we demonstrated that LGK974 could significantly induce apoptosis in ACHN and 786 cell lines by flow cytometry (Figures 4(c) and 4(d)). Then we detected the expression of apoptosis-related proteins Bax and Bcl2. Interestingly, LGK974 upregulated apoptosis protein Bax and downregulated apoptosis inhibitor protein Bcl2 (Figures 4(e) and 4(f)). After drug treatment, the ratio of Bcl2/Bax decreased significantly (Figures 4(e) and 4(f)) which indicated that LGK974 induced apoptosis of ccRCC cells in vitro.

### 3.6. LGK974 Inhibits Migration and Invasion of ccRCC

**Figure 5:**
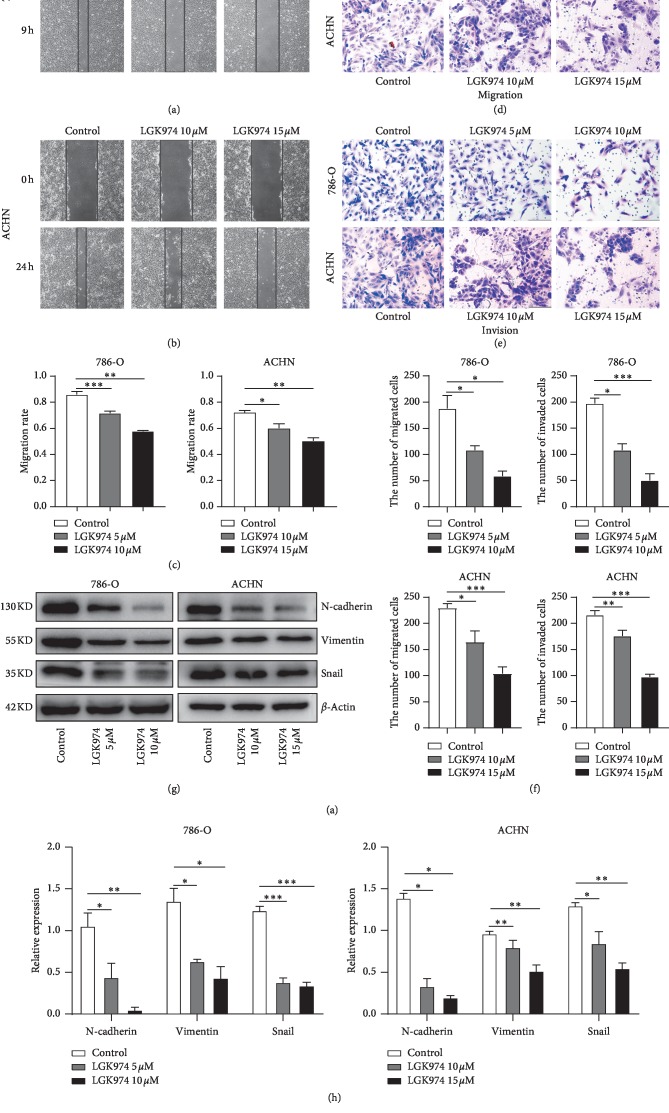
LGK974 inhibits migration and invasion of ccRCC cell lines. (a–c) Cell migration capacity was detected by wound healing assay when renal cancer cells were treated under different concentrations of LGK974. (d–f) Cell migration and invasion capacity were detected by transwell migration and invasion tests when renal cancer cells were treated under different concentrations of LGK974. (g, h) Western blotting experiments were performed by treating renal cancer cells with DMSO, at different concentrations of LGK974 (786-O, 5 *μ*M or 10 *μ*M; ACHN, 10 *μ*M or 15 *μ*M) for 72 h and then proteins were extracted. The protein levels of N-cadherin, vimentin, and snail were determined via immunoblotting. All data are expressed as the mean ± SEM. The experiment was repeated at least three times. Statistical significance was determined using two-tailed Student's *t*-test or one-way ANOVA. ^*∗*^*p* < 0.05; ^*∗∗*^*p* < 0.01; ^*∗∗∗*^*p* < 0.001.

To study whether LGK974 inhibits the ability of ccRCC cells to migrate and invade, we performed wound healing and transwell assay with ACHN and 786-O cells. As shown by the wound healing assay, the results showed LGK974 could greatly inhibit the migration capacity of ACHN and 786-O cells (Figures 5(a)–5(c)). In the transwell assay, we found that LGK974 could significantly inhibit the migration and invasion of renal cancer cells (Figures 5(d)–5(f)). Then we examined the expression of mesenchymal markers in renal cancer cells after LGK974 treatment and the protein expression of N-cadherin, vimentin, and snail. As expected, LGK974 could significantly reduce the protein expression of N-cadherin, vimentin, and snail (Figures 5(g) and 5(h)). During this period, we found that the inhibitory effect of LGK974 on the invasion and migration of ACHN cells was not as obvious as that of 786-O at the same drug concentration.

### 3.7. LGK974 Downregulates Wnt Target Genes and Blocks Cell Cycle

**Figure 6:**
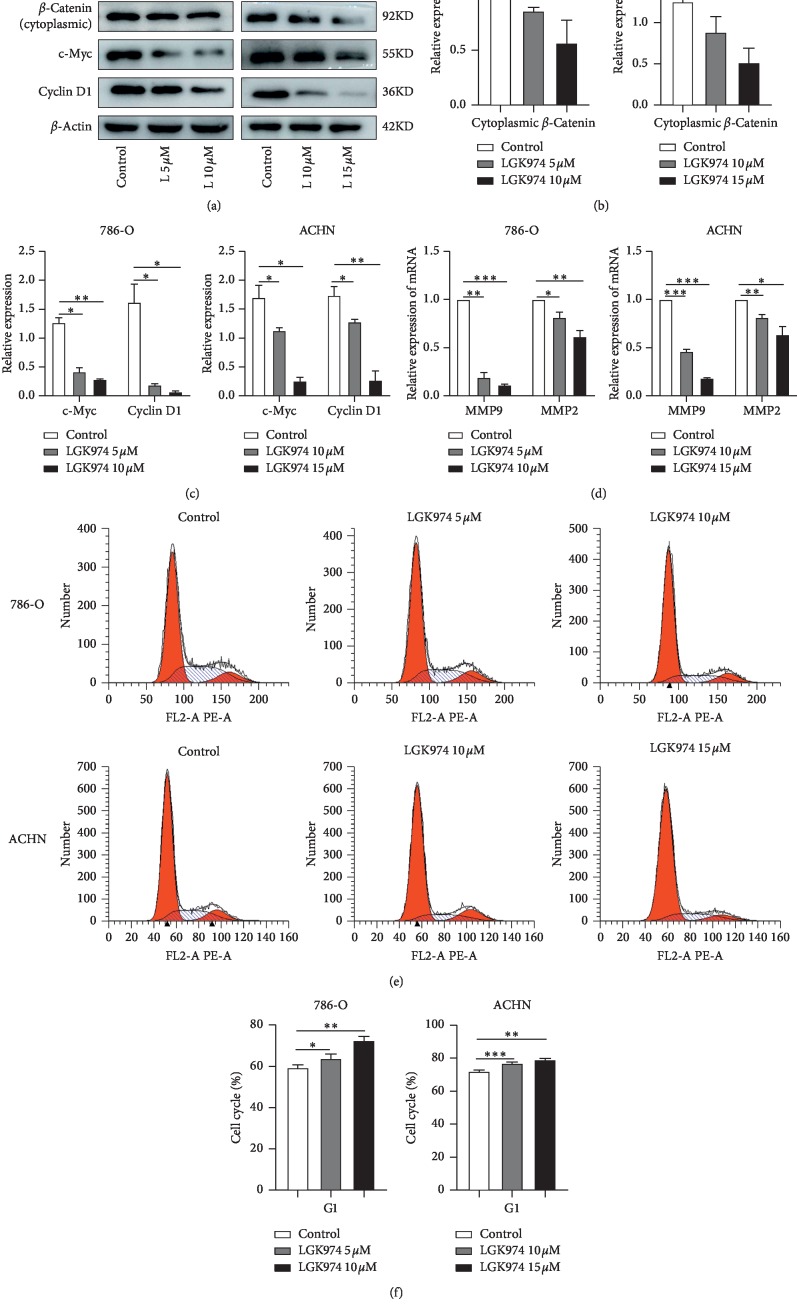
LGK974 downregulates Wnt target genes and blocks cell cycle. (a–c) Western blotting experiments were performed by treating renal cancer cells with DMSO, at different concentrations of LGK974 (786-O, 5 *μ*M or 10 *μ*M; ACHN, 10 *μ*M or 15 *μ*M) for 72 h and then proteins were extracted. The protein levels of *β*-catenin, cyclin D1, and c-Myc were determined via immunoblotting. (d) Real-time PCR experiments were performed by treating renal cancer cells with DMSO, at different concentrations of LGK974 (786-O, 5 *μ*M or 10 *μ*M; ACHN, 10 *μ*M or 15 *μ*M) for 72 h and then total RNA was extracted. (e, f) Cell cycle changes after treating 786-O and ACHN cells with DMSO, at different concentrations of LGK974. All data are expressed as the mean ± SEM. The experiment was repeated at least three times. Statistical significance was determined using two-tailed Student's *t*-test or one-way ANOVA. ^*∗*^*p* < 0.05; ^*∗∗*^*p* < 0.01; ^*∗∗∗*^*p* < 0.001.

As a key molecule of the classical Wnt pathway, *β*-catenin plays an important role in the occurrence and development of cancer. We examined the expression of cytoplasmic *β*-catenin and Wnt target genes in renal cancer cells after treatment with LGK974, including the protein expression level of cyclin D1 and c-Myc and the RNA expression level of MMP9 and MMP2. The results showed that LGK974 could reduce the expression of *β*-catenin in cytoplasm, and LGK974 could significantly reduce the expression of cyclin D1, c-Myc (Figures 6(a)–6(c)), MMP9, and MMP2(Figure 6(d)). Considering that cyclin D1 plays an important role in regulating the cell cycle and according to pathway analysis, PORCN and its related genes will have an effect on the cell cycle. We examined the effect of LGK974 on the cycle of renal cancer cells. The results of cell cycle analysis showed that compared with the control group, the number of 786-O cells and ACHN cells treated with LGK974 in the G1 phase was significantly increased (Figures 6(e) and 6(f)).

## 4. Discussion

Although PORCN has been shown to play an important role in the secretion and activation of Wnt proteins, little is known about its role in renal cell carcinoma. In many other tumors, inhibition of PORCN has a good tumor-suppressing effect.

In lung adenocarcinoma (LUAD), after using the PORCN inhibitor LGK974 or small interfering RNA, the target genes of the Wnt pathway was downregulated, the activity of Wnt pathway was decreased, and the growth of tumor was significantly inhibited [22, 23]. In the breast cancer models driven by MMTV-Wnt1, the application of the PORCN inhibitor LGK974 or Wnt-C59 showed a strong tumor-suppressing effect [15, 24]. In gastric cancer and glioblastoma, PORCN inhibitors significantly inhibit tumor cell proliferation and migration and induce apoptosis [25, 26]. Moreover, the PORCN inhibitor LGK974 also showed significant anticancer effects in combination therapy. In primary ovarian cancer ascites cells, the combination of LGK974 and carboplatin can significantly induce cytotoxicity and cell cycle arrest compared with monotherapy [27], not only that, LGK974 enhance the sensitivity of liver cancer to radiotherapy [28], and the combination of LGK974 and nilotinib (NIL) significantly enhances the ability to inhibit proliferation and colony formation of CML stem cells and progenitor cells and reduces their growth in immunodeficient mice in vivo [29]. More interestingly, in head and neck squamous cell carcinomas (HNSCC), Kleszcz et al. found that PORCN inhibitors not only effectively induce tumor cell apoptosis but also inhibit their migration ability, and in many targets of Wnt pathway, PORCN and CBP are the most significant targets for tumor inhibition [30]. However, the antitumor effect produced by inhibiting PORCN is not obvious in some tumors and even produces a tumor-promoting effect. For example, in Ewing sarcoma (ES), LGK974 inhibits cell migration ability well but does not inhibit tumor cell proliferation and primary tumor growth [31]. Furthermore, Huels et al. found that the use of PORCN inhibitor to reduce the secretion of Wnt ligand accelerated the fixation of APC-deficient cells in the crypt, thereby accelerating tumorigenesis [32].

Unfortunately, the impact of such an interesting gene on kidney cancer has not been systematically studied, and even no studies have been conducted in urinary tumors. In this study, we used bioinformatics analysis and experimental verification to explore the role of PORCN in renal cell carcinoma.

In order to explore the effect of PORCN inhibitor LGK974 on renal cell carcinoma, 786-O and ACHN cell lines were mainly used in our study. VHL mutation was found in 786-O cell line, but there was no VHL mutation in ACHN. ACHN is a metastatic renal cancer cell found in pleural effusion and 786-O is a primary renal cancer cell, so the cell lines we chose are very representative.

We used real-time PCR to detect the expression of PORCN in three cell lines. It was found that the expression of PORCN in 786-O and ACHN was higher than that of normal renal epithelial cells HK-2, especially 786-O, and the expression of PORCN was 17 times higher than that of HK-2. Then we analyzed the difference in survival between patients with high and low expression of PORCN in renal cancer patients by the Human Protein Atlas online tool. We found that patients with low expression of PORCN had significantly better survival than those with high expression of PORCN. Here we can basically determine that PORCN is involved in the development of kidney cancer as an oncogene.

The analysis of the STRING website showed that the 10 proteins most related to PORCN were Wnt proteins, and most of them had varying degrees of mutations in renal cell carcinoma. The results of GO analysis and KEGG analysis suggested that PORCN and its related genes will play a role through the Wnt signaling pathway, which was also illustrated by the interaction of PORCN and its related genes with Wnt pathway-related proteins, apoptosis-related proteins, and EMT-related proteins. This was proved by the results of pathway analysis on the David website and KOBAS online tools and the coexpression of PORCN and Wnt protein again.

In subsequent experiments, we found that PORCN inhibitor LGK974 killed renal cancer cells in a time- and concentration-dependent manner and inhibits renal cancer cell proliferation, induces apoptosis, and inhibits invasion and migration of renal cancer cells. However, the inhibitory effect of LGK974 on ACHN is not as good as 786-O in inhibiting migration and invasion, which may be due to the fact that ACHN is a metastatic cell and conforms to the hypothesis that LGK974 suppresses the early steps of metastasis cascade reaction (such as migration and invasion) [31]. Therefore, we have every reason to believe that PORCN can be used as an effective target to inhibit the occurrence and development of renal cell carcinoma.

**Figure 7:**
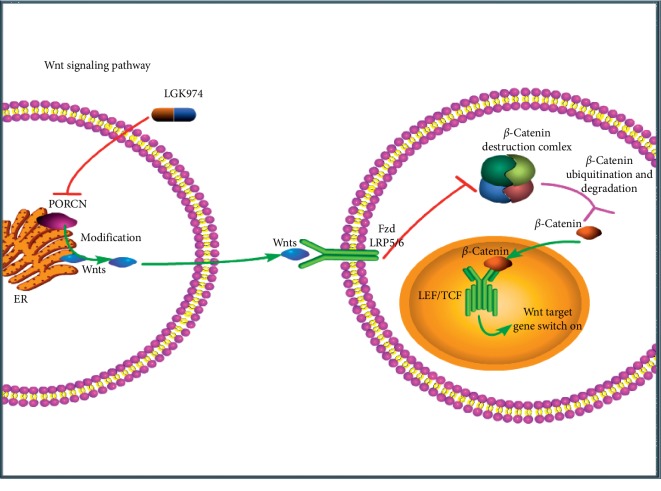
Schematic illustration of LGK974 on ccRCC.

We explored whether LGK974 works by regulating the Wnt signaling pathway. LGK974, as a PORCN inhibitor, has a wide range of effects on the Wnt signaling pathway, and it has an effect on both classical and nonclassical Wnt pathways. Due to the lack of a marker of universal activation of nonclassical Wnt pathways, it is difficult to detect the activity of all nonclassical Wnt pathways, so we focused on the effect of LGK974 on the activity of Wnt/*β*-catenin signaling pathway. As a classical marker of Wnt signaling pathway, the expression level of *β*-catenin in cytoplasm (cytoplasmic *β*-catenin rather than nuclear *β*-catenin was identified as most associated with adverse clinicopathology and poor prognosis [11]) decreased significantly, and the expression levels of target genes c-Myc, cyclin D1, MMP9, and MMP2 in Wnt signaling pathway decreased significantly. The mechanism diagram is shown in Figure 7.

## 5. Conclusions

Taken together, we demonstrated that the expression of PORCN was upregulated in renal cancer cell lines. PORCN inhibitor LGK974 can inhibit cell proliferation, migration, and invasion, induce apoptosis, and block cell cycle. Further analysis showed that LGK974 could downregulate the activity of the Wnt/*β*-catenin signaling pathway. These data also provide evidence for the feasibility of targeting PORCN to downregulate the Wnt/*β*-catenin signaling pathway in renal cell carcinoma and reveal the possibility of PORCN inhibitors as potential targets for the treatment of renal cell carcinoma.

## Data Availability

The data used to support the findings of this study are available from the corresponding author upon request.
